# Risk and outcome of subsequent malignancies after radioactive iodine treatment in differentiated thyroid cancer patients

**DOI:** 10.1186/s12885-021-08292-8

**Published:** 2021-05-13

**Authors:** Xiaoran Mei, Xiaoqin Yao, Fang Feng, Weiwei Cheng, Hui Wang

**Affiliations:** 1 Xin Hua Hospital Affiliated to Shanghai Jiao Tong University School of Medicine, Shanghai, China; 2 The Affiliated Yantai Yuhuangding Hospital of Qingdao University, Yantai, China

**Keywords:** Radioactive iodine, Subsequent malignancies, Differentiated thyroid cancer

## Abstract

**Background:**

We identified differentiated thyroid cancer (DTC) survivors from SEER registries and performed Poisson regression to calculate the relative risks (RRs) of subsequent malignancies (SMs) by different sites associated with radioactive iodine (RAI) treatment, and the attributable risk proportion of RAI for developing different SMs.

**Results:**

We identified 4628 of 104,026 DTC patients developing a SM after two years of their DTC diagnosis, with a medium follow-up time of 113 months. The adjusted RRs of developing SM associated with RAI varied from 0.98 (0.58–1.65) for neurologic SMs to 1.37 (1.13–1.66) for hematologic SMs. The RRs of developing all cancer combined SMs generally increased with age at DTC diagnosis and decreased with the latency time. We estimated that the attributable risk proportion of RAI treatment is only 0.9% for all cancer combined SMs and 20% for hematologic SMs, which is the highest among all SMs. The tumor features and mortalities in patients treated with and without RAI are generally comparable.

**Conclusion:**

With the large population based analyses, we concluded that a low percentage of DTC survivors would develop SMs during their follow-up. Although the adjusted RR of SMs development increased slightly in patients receiving RAI, the attributable risk proportion associated with RAI was low, suggesting the absolute number of SMs induced by RAI in DTC survivors would be low. The attributable risk proportion of RAI treatment is the highest in hematological SMs, but when in consideration of its low incidence among all DTC survivors, the absolute number of hematological SMs was low.

**Supplementary Information:**

The online version contains supplementary material available at 10.1186/s12885-021-08292-8.

## Introduction

Differentiated thyroid cancer (DTC) is the most prevalent endocrine cancer and the incidence of DTC has increased dramatically worldwide in the past few decades [1, 2]. Generally, DTC patients have a favorable prognosis after appropriate treatment, with the 10-year survival rate estimated to be greater than 90% [3, 4]. Given the rising incidence and the good prognosis of DTC, the development of a subsequent malignancy (SM) became an important concern for DTC survivors and also physicians [5–7]. It has been reported that DTC survivors have an 10–30% higher risk of developing a SM comparing with the general population [5–8]. These greater risks are probably a result of the combination of lifestyle, environment, genetic factors and the medical treatment for DTC.

Radioactive iodine (RAI) is commonly used in DTC treatment [9]. In a recent multicenter cohort study, there were 57.5% DTC patients received RAI during the initial treatment of primary tumor, lower than 62–75% reported by National Thyroid Cancer Treatment Co-operative Group based on patients diagnosed between 1987 to 2001 [10, 11]. Increased number of studies support to reduce unnecessary radioiodine treatment in DTC patients in the last decades [12, 13]. However, the balance of benefits and risks of RAI treatment in DTC patients are still inconclusive yet. While most studies reported RAI treatment is associated with an increased risk of SMs development in DTC survivors [5–8, 14], there are also some investigations suggesting a minor effect of RAI in inducing SMs [15, 16]. In addition, analyses basing on all adult cancer survivors have indicated that most SMs are actually developed due to non-radiation factors, such as lifestyle or genetics, and concluded that a small proportion of SMs (< 10%) might be truly related to radiotherapy [17, 18]. Among all these studies investigating the risks of SMs in DTC survivors, none of them regarding the attributable risk of RAI treatment in inducing SMs development. Moreover, very few studies compared the biological features of SMs in RAI treated and non-RAI treated DTC survivors, as well as the clinical outcome of the two groups of patients.

Therefore, by using data from the SEER registries, we systemically investigated the proportion of SMs might be induced by RAI treatment in DTC survivors in this study, and also compared the biological features of SMs and the mortality of DTC survivors treated with and without RAI. Our analyses would provide supplemental evidences towards the application of RAI treatment in DTC patients.

## Methods

### Data source and participants

The cohort was assembled using the April 2020 release of all 18 registries of the SEER database which covered approximately 28% of the US population. As the spectrum of pediatric and adolescent tumor is different from that of adults, only patients aged 20 years or older who were diagnosed with a first primary thyroid cancer of papillary or follicular type between 2000 and 2016 (the histological subtypes included in analysis were ICD codes 8050, 8260, 8290, 8330–8332, 8335, 8340–8344 and 8350) were identified by the SEER program statistical analysis software (SEER*Stat, version 8.3.6). We used the variant “summary stage” to define the extent of DTC. As there is a lag time between radiation exposure and SM development, we exclude patients whose follow-up time were less than 24 months after their diagnosis of thyroid cancer. This criterion also ensured that we minimized the surveillance bias that might generated when patients who received RAI treatment were monitored more intensive than those not in the first 24 months. Our study did not need ethics committee approval because the data are publicly available.

### Procedures and statistical analysis

The SEER*Stat MP-SIR (Multiple Primary-Standardized Incidence Ratio) tool was used to extract the details of all included DTC survivors. We used Poisson regression analysis to estimate the relative risks (RRs) with 95% CIs and *P* values of SMs development in DTC survivors who received RAI compared with those who did not. The RRs were estimated for all combined SMs and also for different SMs by their sites, and further adjusted with age at DTC diagnosis, gender, year of DTC diagnosis and tumor stage. The RRs were also estimated in subgroup patients stratified according to their gender, age at DTC diagnosis and latency time from DTC diagnosis to SM diagnosis. The number of excess SMs related to RAI treatment was calculated by taking the number of SMs in those treated with RAI minus the estimated number of SMs in these patients if they were not treated with RAI. Attributable risks were also assessed for different SMs by their sites, which quantify the risk in RAI treated DTC survivors that was attributable to RAI treatment.

The statistical analysis was performed similarly as our previous work [19]. To be specific, categorical data were summarized as frequencies and percentages while continuous data were summarized as medians and interquartile ranges (IQR). The Chi-Squared Test was used to analyze categorical variables while Wilcoxon-Mann-Whitney test was used to analyze continuous variables. All statistical analyses were performed using SPSS (version 22). Statistical significance was defined as a *P* value of less than 0.05, all statistical tests were two sided.

## Results

### Patient characteristics

We identified 104,026 patients with DTC from the SEER database in total, 51,212(49.2%) patients received RAI as part of their DTC treatment while 52,814(50.8%) patients did not. Basic demographic and disease characteristics of these DTC patients are shown in Table 1. RAI treated DTC patients tend to have lower percentage of females, younger age, and higher stage of tumors.

**Table 1:** Characteristics of patients with differentiated thyroid cancer enrolled in this study

	+RAI	-RAI	***P value***
**No. of patients, n**	51,212	52,814	
**Patients with SMs, n(%)**	2289(4.5%)	2339(4.4%)	*0.752*
**Gender**			*< 0.001*
**Female**	38,941	42,423	
**Male**	12,271	10,391	
**Median age at diagnosis of thyroid cancer (IQR), yrs**	46(36–56)	49(38–59)	*< 0.001*
**Race**			*< 0.001*
**White**	41,771	42,949	
**Black**	2944	3983	
**Asian and Pacific Islander**	5825	4816	
**American Indian/Alaska native**	275	232	
**Unknown**	397	834	
Stage*			*< 0.001*
**Localized**	29,943(58.5%)	43,872(83.1%)	
**Regional**	19,304(37.7%)	6987(13.2%)	
**Distant**	1650(3.2%)	635(1.2%)	
**Unknown/unstaged**	315(0.6%)	1320(2.5%)	
**Median follow-up time (IQR), months**	91(57–133)	85(52–130)	*< 0.001*

*: Localized: lesions confined to thyroid; Regional: regional by direct extension or/and regional lymph node involved; Distant: distant site(s)/lymph node involved

During the follow-up period (2000–2016), a total of 4628 (4.4%) DTC survivors developed SMs. Among them, 2289 patients have received RAI treatment (RAI+) and 2339 patients have not (RAI-), the incidences of all cancer combined SMs in RAI+ and RAI- DTC survivors were 4.5% versus 4.4%, showing no difference(*P = 0.752*). Breast cancer is the most common SM in all DTC survivors (1.1%) while the lowest incidence of SMs are neurologic cancer (0.06%) (Table S1). The spectrum of different SMs was highly overlapped between the two groups (Fig. S1). Exceptions were cancers of digestive and hematologic system, for which SMs of digestive system accounts for a lower percentage (13.9% vs 15.2%, *P = 0.216*) and SMs of hematologic system accounts for a higher percentage (11.4% vs 9.2%, *P = 0.013*) in RAI+ group, as compared to RAI- group. The descriptive statistics of different SMs in RAI+ and RAI- patients were listed in Table 2.

**Table 2:** Descriptive statistics of the SMs in DTC survivors by their treatment

	Number of patients, n(%)		Number of Females, n(%)		Proportion of patients by race(W/B/API)		Proportion of patients by disease stage (Local/Regional/Distant)		Median age at diagnosis of the SMs, yrs		Median follow-up time since the diagnosis of SMs,months		Median latency time, months	
	+RAI	-RAI	+RAI	-RAI	+RAI	-RAI	+RAI	-RAI	+RAI	-RAI	+RAI	-RAI	+RAI	-RAI
**All cancer combined**	**2289**	**2339**	**68.7%**	**72.6%**	**83%/5%/11%**	**85%/8% /0%**	**50%/18% /17%**	**50%/21% /16%**	**62**	**64**	**28**	**26**	**70**	**70**
Oral Cavity and Pharynx	56	51	64.3%	64.7%	79%/5% /16%	78%/12%/10%	48%/11% /7%	39%/12% /8%	58	59	22	24	68	73
Digestive System	319	356	69.0%	72.5%	78%/8% /14%	81%/10%/10%	36%/28% /24%	34%/32% /4%	64	65	19	14	73	68
Respiratory System	247	254	68.8%	72.4%	82%/5% /12%	83%/9% /8%	24%/17% /52%	24%/24% /48%	68	66	14	10	68	66
Skin	138	137	63.0%	67.2%	96%/1% /4%	96%/1% /2%	76%/9% /3%	78%/11% /1%	56	64	35	31	65	68
Breast	547	574	99.5%	99.8%	80%/5% /15%	82%/10%/8%	65%/30% /4%	66%/30% /3%	58	61	35	34	73	74
Female Genital System	179	206	100.0%	100.0%	83%/7% /10%	82%/5% /12%	61%/16% /16%	51%/23% /21%	60	62	29	26	66	67
Male Genital System	237	233	0.0%	0.0%	84%/7% /9%	88%/9% /3%	79%/14% /3%	82%/12% /2%	65	66	38	50	70	69
Urinary System	194	191	43.3%	57.6%	89%/3% /8%	90%/5% /6%	55%/12% /10%	56%/9% /8%	64	67	30	31	71	78
Nervous System	30	36	80.0%	69.4%	87%/7% /7%	94%/3% /0%	73%/20% /3%	86%/8% /0%	57	60	15	12	55	60
Hematologic System	261	215	65.1%	65.6%	84%/4% /11%	87%/7% /4%	NA	NA	63	66	27	23	65	73

### Relative risk of developing SMs associated with RAI treatment

**Fig. 1:**
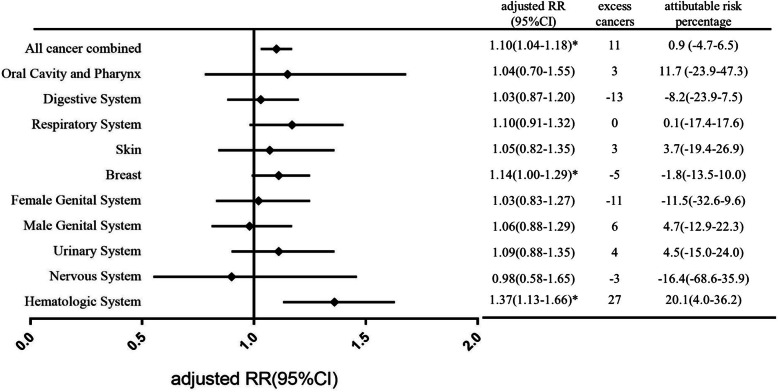
The adjusted relative risk of SMs, the estimated number of excess cancers and the attributable risk associated with RAI treatment by the site of SMs. The RR was adjusted with age at DTC diagnosis, gender, year of DTC diagnosis and tumor stage

To investigate how much RAI treatment attribute to the increased risk of SMs development, we assessed the crude and adjusted RRs of SMs associated with RAI treatment (Fig. 1 and Table S2). The significant increased RR was only seen for hematologic cancers (RR = 1.25, 95%CI: 1.05–1.50; *P = 0.015*), and it became more significant after adjusting with age at DTC diagnosis, gender, year of DTC diagnosis and tumor stage (RR = 1.37, 95%CI:1.13–1.66; *P = 0.001*). Adjustment for age at DTC diagnosis, gender, year of DTC diagnosis and DTC tumor stage have a small effect on the RR estimates, generally increasing the risks (Fig. 1 and Table S2). The crude RRs for all cancer combined SMs and breast cancers were 1.01 (95%CI:0.95–1.07, *P = 0.749*) and 0.98 (95%CI:0.87–1.11, *P = 0.778*), and increased to 1.10 (95%CI:1.03–1.17, *P = 0.003*) and 1.14(95%CI:1.00–1.29, *P = 0.040)* respectively after adjustment, which became statistically significant (Fig. 1 and Table S2).

After defining a latency time of two years between DTC diagnosis and SM development, there were an estimated 11 excess SMs for all cancer combined that could be related to RAI treatment in our analysis (Fig. 1). The attributable risk of RAI treatment for SMs development was only 0.9%(95%CI: − 4.7-6.5%), indicating RAI treatment only contribute little in inducing SMs development in DTC survivors. This proportion was relatively high in patients developed SMs in hematologic system and oral cavity and pharynx system, for which 20.1 and 11.7% SMs could be related to RAI treatment, respectively (Fig. 1). This is consistent with other studies reporting RAI is more likely to induce leukemia and salivary gland malignancies [5, 6, 16].

We additionally assessed effect modification by age, gender and latency time between the diagnosis of DTC and SM. The RRs of SMs development associated with RAI treatment gradually increase with the age at DTC diagnosis, although the significant increase was only seen in patients with their DTC diagnosed between 60 and 74 yrs. (RR = 1.17,95%CI:1.05–1.30, *P = 0.004*) (Table 3). By contrast, the RRs for hematologic SMs were significantly increased in all age subgroups, excepting patients with DTC diagnosed older than 75 yrs. (Table 3). Both genders were not at the increased risk of all cancer combined SMs associated with RAI treatment, but females receiving RAI had an increased risk of hematologic SMs compared to those did not receive RAI (RR = 1.31,95%CI:1.05–1.64, *P = 0.016*). The RRs of SMs relative to RAI treatment were significantly elevated in the first 5 years after DTC diagnosis (all cancer combined: RR = 1.11, 95%CI:1.01–1.22, *P = 0.025*; hematologic cancers: RR = 1.83, 95%CI:1.14–2.94, *P = 0.012*) and gradually decreased with increasing time since DTC diagnosis (Table 3).

**Table 3:** Relative risk of all SMs or hematologic SMs for RAI therapy by different stratification

	All cancer combined		Hematologic SMs	
Stratified by diagnosed age	RR(95%CI)	*P value*	RR(95%CI)	*P value*
< 45 yrs	1.05(0.92–1.21)	*0.454*	2.17(1.08–4.38)	*0.035*
45-59 yrs	1.08(0.99–1.19)	*0.094*	1.50(1.06–2.10)	*0.02*
60-74 yrs	1.17(1.05–1.30)	*0.004*	1.70(1.29–2.25)	*0.000*
> =75 yrs	1.18(0.96–1.46)	*0.123*	1.04(0.69–1.58)	*0.832*
**Stratified by gender**				
female	1.01(0.94–1.08)	*0.816*	1.31(1.05–1.64)	*0.016*
male	0.94(0.85–1.05)	*0.303*	1.04(0.76–1.41)	*0.814*
**Stratified by latency time**				
< 5 yrs	1.11(1.01–1.22)	*0.025*	1.83(1.14–2.94)	*0.012*
5-10 yrs	1.03(0.94–1.13)	*0.536*	1.13(0.85–1.50)	*0.413*
10-15 yrs	0.88(0.76–1.02)	*0.086*	1.19(0.88–1.62)	*0.266*
> =15 yrs	0.75(0.43–1.33)	*0.325*	0.97(0.56–1.67)	*0.898*

### Comparison of SMs between RAI-treated and non RAI treated DTC survivors

Although RAI+ patients tend to be diagnosed with DTC of higher tumor stage, the features of SMs and the outcome of patients are overally comparable in RAI+ and RAI- patients (Tables 2 and 4). The descriptive characteristics of SMs between two groups of patients are presented in Table 2. DTC patients receiving RAI treatment tend to have higher tumor stage (Table 1), but the proportion of patients developed SMs are comparable in RAI+ and RAI- group (Table 2), indicating tumor stage has little effect on the relative risk of SMs development associated to RAI treatment. There are a few exceptions: a longer follow-up time were seen in RAI+ patients developing SMs of skin and in RAI- treated patients developing SMs of male genital system; and a lower mortality in RAI treated patients developing SMs of digestive system. DTC survivors treated with RAI tend to have a lower overall mortality and disease specific mortality (death caused by SPM), although no statistically significance (Table 4).

**Table 4:** The mortality of DTC survivors developing SMs by different sites

	overall mortality			disease specific mortality		
	with RAI	no RAI	*P value*	with RAI	no RAI	*P value*
All cancer combined	25.5%	28.0%	*0.001*	16.4%	18.3%	*0.081*
Oral Cavity and Pharynx	26.8%	23.5%	*0.008*	7.1%	3.9%	*0.681*
Digestive System	39.5%	47.2%	*0.052*	32.3%	36.8%	*0.225*
Respiratory System	62.3%	61.0%	*0.783*	47.8%	49.2%	*0.789*
Skin	12.3%	11.7%	*1.000*	5.1%	2.9%	*0.54*
Breast	9.9%	11.0%	*0.559*	4.8%	5.9%	*0.427*
Female Genital System	20.1%	22.8%	*0.537*	14.5%	19.4%	*0.224*
Male Genital System	8.4%	9.0%	*0.871*	3.4%	3.4%	*1.000*
Urinary System	17.5%	19.9%	*0.602*	10.3%	8.9%	*0.73*
Nervous System	63.3%	66.7%	*0.801*	60.0%	63.9%	*0.802*
Hematopoietic System	25.7%	29.3%	*0.409*	17.2%	20.9%	*0.347*

## Discussion

As the good prognosis of DTC, developing a SM is probably the greatest concern in DTC survivors [20]. Indeed, SM has been reported as a major cause of mortality and serious morbidity in DTC survivors. Compared to the general population, DTC survivors have a 10–30% higher risk of developing a SM [5–8], due to the genetic predisposition, environmental factors, lifestyle, and the cancer treatment they received. RAI is commonly used in DTC treatment for three purposes: 1) RAI remnant ablation to facilitate detection of recurrent disease in the surveillance with serum thyroglobulin; 2) RAI adjuvant therapy to eliminate suspected residual disease; 3) RAI therapy to treat persistent disease [9, 21]. Many evidences have shown that RAI treatment can decrease the metastasis and improve the survival of DTC patients [22–24]. For low- and intermediate-risk patients, RAI treatment is gradually questioned in the recent few years, as some studies indicated these patients have relative good prognosis, but will risk themselves to develop a SM if receiving RAI treatment [6–8, 16, 25]. However, extensive oppositions existed [26–29]. Considerable arguments about the balance between benefits and harms, as well as the quality of patient care, were generated and widely spread in physicians, especially in the nuclear medicine community [26–29].

The risk of SMs development associated with RAI in DTC survivors have been investigated and debated for decades [5–8, 16, 30]. Many studies presented their evaluations in a way may be interpreted by statisticians, but not the majority of clinical physicians, who will really read these statistics with the goal of weighting the pros and cons of RAI treatment in their patients. Therefore, in this study, we comprehensively analyzed the risk associated with RAI, also compared the clinical features of SMs as well as the mortality of RAI+ and RAI- treated patients. More specifically, we estimated the proportion of SMs risk directly associated with RAI, which for the first time quantitatively showed the absolute risk of RAI in inducing SMs. These data can be more easily and intuitively interpreted by physicians and patients. Our main findings include: 1) The adjusted RR associated with RAI was only significantly increased for SMs of hematologic systems and breast; 2) Only 0.9% of all cancer combined SMs are estimated to be attributed to RAI treatment; the proportion is relatively high in patients developing SMs in hematologic systems and oral cavity and pharynx system (20.1 and 11.7% respectively);3) The RRs of all cancer combined SMs associated with RAI generally increased with age at DTC diagnosis and decreased with the latency time; by contrast, the RRs of hematologic SMs peaked in patients with DTC diagnosed younger than 45 yrs., and then decreased with age at DTC diagnosis; 4) The clinical features and mortality are overally comparable between RAI+ and RAI- patients.

Many studies have claimed that RAI would associate with a risk of SM development as its carcinogenesis effect, but the real concern is how much the risk is and how the absolute number is. In this study, we estimated that the attributable risk of RAI treatment for all cancer combined SMs was only 0.9%. Given the relative low incidence of SMs in DTC survivors (4.4%) and this small attributable risk proportion of RAI treatment, the absolute number of SMs associated with RAI treatment in DTC survivors would be low. Hematologic system is the most susceptible system to develop SMs after RAI treatment, the adjusted RR is 1.37(95%CI:1.13–1.66, *P = 0.001*) and the attributable risk proportion of RAI treatment is around 20%, which is the highest among all cancers. However, the incidence of hematologic SMs in all DTC survivors is only 0.46%, indicating the absolute number would be low. In addition, there is no way to exclude the effect of hyperthyroidism on the hematologic SMs development. RAI treated patients usually are in iatrogenic subclinical hyperthyroidism, which has been reported as an independent risk factor for leukemia [27, 31]. Taken together with these factors, we think both physicians and patients should be rational about the risk associated with RAI in inducing hematologic SMs.

Although patients receiving RAI treatment tend to have higher stage of DTC tumor, the clinical features of SMs are comparable between RAI+ and RAI- patients, indicating neither RAI treatment nor the intrinsic biological aggressiveness of DTC tumor would affect the clinical feature of SMs. Consistent with the clinical features, SM specific mortalities are also comparable in two groups of patients. However, the overall mortalities tend to be lower in RAI treated patients, with statistical significance for all cancer combined SMs. Surveillance bias cannot be ruled out for the lower mortality, as indolent malignancies are more likely to be discovered during the frequent surveillances in RAI+ patients and/or these patients are more likely to change their lifestyle due to their more advanced tumors. Other factors interacting with RAI may also contribute to the lower mortality.

The main limitation of the SEER data is lacking the amounts of administered activities of RAI, therefore, it is not possible to determine the dose-response effect of RAI in this study. Some studies with available information observed an increased leukemia risk associated with RAI, but only with a dosage higher than 100 mCi or even 150mCi [16, 25]. This means only a small percentage of patients, who accept RAI activity that above the most commonly used dosage (50-100 mCi), should be concern of this increased risk. However, in the other hand, patients receiving this high amount of dosage usually have advanced tumors in which RAI has shown survival advantage [9]. Therefore, it is critical to weight the benefits and harms of RAI treatment, and determine the dosage of RAI in each individual DTC survivors in clinical practice.

In summary, in this population based data analysis, we found only for SMs of hematologic and breast SMs, RAI treatment is associated with an increased RRs. For all cancer combined SMs, a low proportion is associated with RAI treatment. Only for hematologic SMs, RAI treatment accounts for a relative high attributable risk proportion. In consideration of the low incidence of SMs in all DTC survivors, the absolute number of SMs in DTC patients would be small, including hematologic SMs. Tumor features and the mortality of RAI+ and RAI- patients are comparable. Taken all these together, we think it is important to provide the most careful assessment of risks and benefits of RAI to each individual patient in clinical practice, but should not be panicked by the potential risk of SMs.

## Supplementary Information

**Additional file 1 Fig. S1.** The spectrum of SMs in patients treated with and without RAI. The x axis shows different cancer types by their sites while y axis shows the percentage of different SMs among all cancer combined. Except for cancers of digestive and hematologic system, the spectrum of different SMs in the two groups (blue line: treated with RAI; red line: not treated with RAI) are overally similar. **Table S1.** The incidence of SMs in all DTC survivors and the proportion of each system of SMs by their sites. The second column (Incidence in all DTC survivors) shows the incidence of different SMs among all DTC survivors (104,026 patients in total), the third and fourth column shows the number (proportion in all SMs) of different SMs by their sites. **Table S2.** The crude relative risk of SMs associated with RAI treatment by the site of SMs.

## Data Availability

The datasets analyzed during the current study available from the corresponding author on reasonable request.
