# Supplementary material for: Risk and outcome of subsequent malignancies after radioactive iodine treatment in differentiated thyroid cancer patients
Source: BMC Cancer. 2021 May 13;21:543. doi: 10.1186/s12885-021-08292-8 (PMC8117631; doi:10.1186/s12885-021-08292-8)
Supplement: Supplementary file 1 — Additional file 1 Fig. S1. The spectrum of SMs in patients treated with and without RAI. The x axis shows different cancer types by their sites while y axis shows the percentage of different SMs among all cancer combined. Except for cancers of digestive and hematologic system, the spectrum of different SMs in the two groups (blue line: treated with RAI; red line: not treated with RAI) are overally similar. Table S1. The incidence of SMs in all DTC survivors and the proportion of each system of SMs by their sites. The second column (Incidence in all DTC survivors) shows the incidence of different SMs among all DTC survivors (104,026 patients in total), the third and fourth column shows the number (proportion in all SMs) of different SMs by their sites. Table S2. The crude relative risk of SMs associated with RAI treatment by the site of SMs. [file 12885_2021_8292_MOESM1_ESM.docx]

**Supplemental Data**

Risk and outcome of subsequent malignancies after radioactive iodine treatment in differentiated thyroid cancer patients

Xiaoran Mei, Xiaoqin Yao, Fang Feng, Weiwei Cheng, Hui Wang


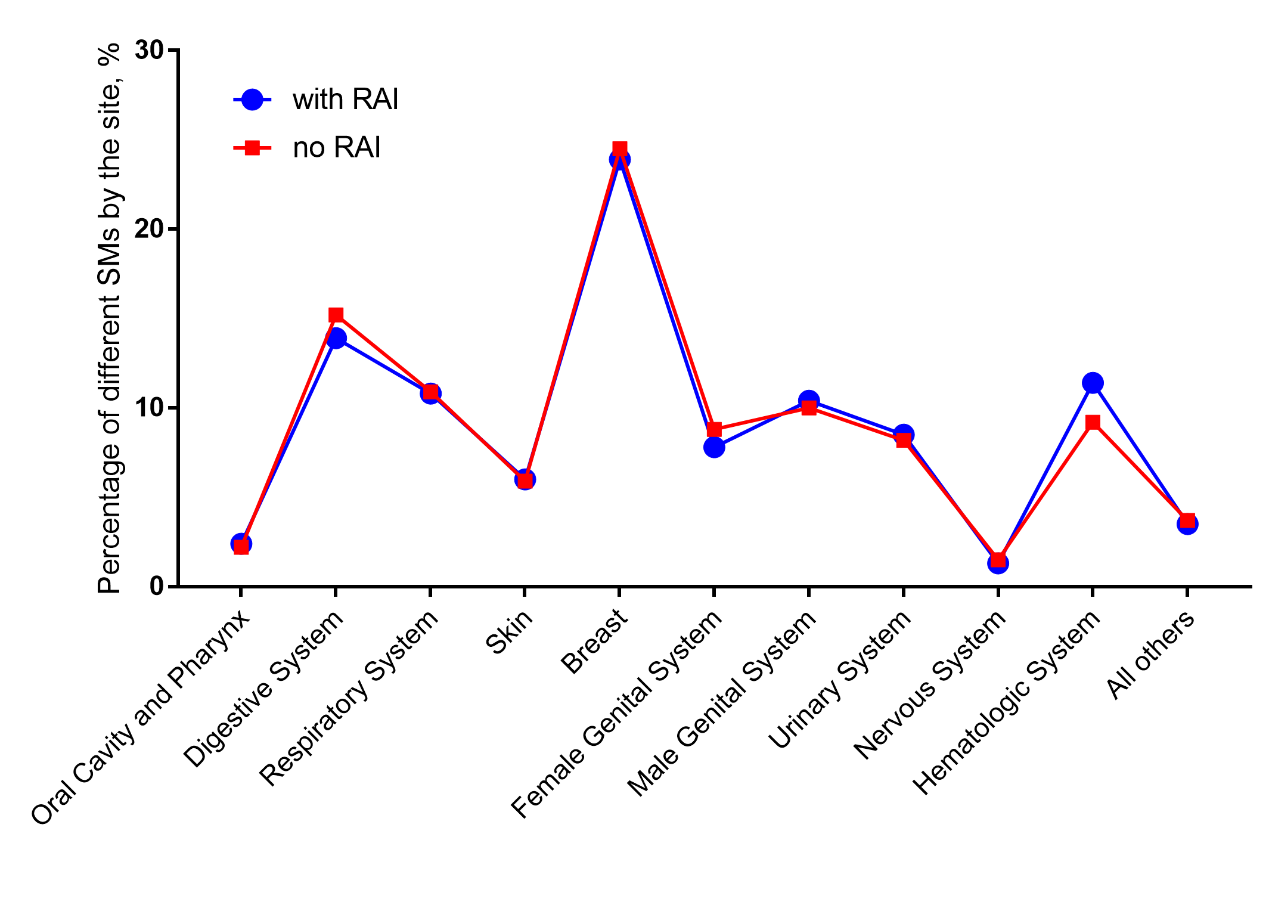
Figure S1. The spectrum of SMs in patients treated with and without RAI. The x axis shows different cancer types by their site while y axis shows the percentage of different SMs among all cancer combined. Except for cancers of digestive and hematologic system, the spectrum of different SMs in the two groups (blue line: treated with RAI; red line: not treated with RAI) are overally similar.

|  | Incidence in all DTC survivors | Number of patients, n(%) | |
| --- | --- | --- | --- |
|  |  | +RAI | -RAI |
| **All cancer combined** | **4.47%** | **2289** | **2339** |
| Oral Cavity and Pharynx | 0.11% | 56(2.4%) | 51(2.2%) |
| Digestive System | 0.62% | 319(13.9%) | 356(15.2%) |
| Respiratory System | 0.48% | 247(10.8%) | 254(10.9%) |
| Skin | 0.27% | 138(6.0%) | 137(5.9%) |
| Breast | 1.07% | 547(23.9%) | 574(24.5%) |
| Female Genital System | 0.35% | 179(7.8%) | 206(8.8%) |
| Male Genital System | 0.46% | 237(10.4%) | 233(10.0%) |
| Urinary System | 0.37% | 194(8.5%) | 191(8.2%) |
| Nervous System | 0.06% | 30(1.3%) | 36(1.5%) |
| Hematologic System | 0.51% | 261(11.4%) | 215(9.2%) |

Table S1. The incidence of SMs in all DTC survivors and the proportion of each system of SMs by their site. The second column (Incidence in all DTC survivors) shows the incidence of different SMs among all DTC survivors (104,026 patients in total), the third and fourth column shows the number (proportion in all SMs) of different SMs by their site.

|  | **Crude RR (95% CI)** | ***P value*** |
| --- | --- | --- |
| All cancer combined | 1.01(0.95-1.07) | *0.749* |
| Oral Cavity and Pharynx | 1.13(0.78-1.66) | *0.520* |
| Digestive System | 0.92(0.79-1.08) | *0.310* |
| Respiratory System | 1.00(0.84-1.20) | *0.971* |
| Skin | 1.04(0.82-1.31) | *0.750* |
| Breast | 0.98(0.87-1.11) | *0.778* |
| Female Genital System | 0.90(0.73-1.10) | *0.286* |
| Male Genital System | 1.05(0.88-1.26) | *0.602* |
| Urinary System | 1.05(0.86-1.28) | *0.647* |
| Nervous System | 0.86(0.53-1.40) | *0.541* |
| Hematologic System | 1.25(1.05-1.50) | *0.015* |

Table S2. The crude relative risk of SMs associated with RAI treatment by the site of SMs.
